# PRCTC: a machine learning model for prediction of response to corticosteroid therapy in COVID-19 patients

**DOI:** 10.18632/aging.203819

**Published:** 2022-01-12

**Authors:** Yue Gao, Xiaoming Xiong, Xiaofei Jiao, Yang Yu, Jianhua Chi, Wei Zhang, Lingxi Chen, Shuaicheng Li, Qinglei Gao

**Affiliations:** 1 Cancer Biology Research Center, Key Laboratory of Chinese Ministry of Education, Tongji Hospital, Tongji Medical College, Huazhong University of Science and Technology, Wuhan 430000, China; 2 Department of Gynecology and Obstetrics, Tongji Hospital, Tongji Medical College, Huazhong University of Science and Technology, Wuhan 430000, China; 3 Department of Computer Science, City University of Hong Kong, Kowloon Tong 999077, Hong Kong

**Keywords:** COVID-19, corticosteroid, machine learning, lymphocyte percent, C reactive protein

## Abstract

Corticosteroid has been proved to be one of the few effective treatments for COVID-19 patients. However, not all the patients were suitable for corticosteroid therapy. In this study, we aimed to propose a machine learning model to forecast the response to corticosteroid therapy in COVID-19 patients. We retrospectively collected the clinical data about 666 COVID-19 patients receiving corticosteroid therapy between January 27, 2020, and March 30, 2020, from two hospitals in China. The response to corticosteroid therapy was evaluated by hospitalization time, oxygen supply duration, and the outcomes of patients. Least Absolute Shrinkage and Selection Operator (LASSO) was applied for feature selection. Five prediction models were applied in the training cohort and assessed in an internal and an external validation dataset, respectively. Finally, two (C reactive protein, lymphocyte percent) of 36 candidate immune/inflammatory features were finally used for model development. All five models displayed promising predictive performance. Notably, the ensemble model, PRCTC (prediction of response to corticosteroid therapy in COVID-19 patients), derived from three prediction models including Gradient Boosted Decision Tree (GBDT), Neural Network (NN), and logistic regression (LR), achieved the best performance with an area under the curve (AUC) of 0.810 (95% confidence interval [CI] 0.760–0.861) in internal validation cohort and 0.845 (95% CI 0.779–0.911) in external validation cohort to predict patients’ response to corticosteroid therapy. In conclusion, PRCTC proposed with universality and scalability is hopeful to provide tangible and prompt clinical decision support in management of COVID-19 patients and potentially extends to other medication predictions.

## INTRODUCTION

Coronavirus disease 2019 (COVID-19), caused by the infection of severe acute respiratory syndrome coronavirus 2 (SARS-CoV-2) [1], has become a global pandemic and brought a heavy burden on health care around the world. As of December 10, 2021, SARS-CoV-2 has led to more than 265 million infections and over five million deaths in 223 countries and territories [2]. Mild COVID-19 patients are often accompanied by fever, cough, and myalgia. However, acute respiratory distress syndrome (ARDS) and multiple organ failure (MOF) are common in severe and critical patients, contributing to the principal cause of death in COVID-19 [3]. A previous study has reported that immune dysregulation and inflammatory cytokines storm played important roles in ARDS and MOF in COVID-19 patients [4]. It is acknowledged that once entering into cells, SARS-CoV-2 will trigger antiviral responses of the hosts' innate and adaptive immunity [5]. For example, numerous immune and inflammatory cells are activated and produce various proinflammatory cytokines, termed cytokine storms. These cytokines will further induce tissue damage which contributes to more production of cytokines in turn. This positive feedback loop finally gives rise to ARDS and MOF [6]. Thus, anti-inflammatory/immunomodulatory therapy may be beneficial especially for severe and critical COVID-19 patients.

Corticosteroid, a kind of affordable, nonspecific anti-inflammatory, and immunomodulatory drug, has been widely applied to treat COVID-19 patients. However, the effect of corticosteroid therapy is an enduring controversy, especially in critical COVID-19 patients [7, 8]. Several published clinical trials revealed that administration of systemic corticosteroids was associated with a lower 28-day mortality compared with usual care or placebo in these patients [9, 10]. But some other studies demonstrated corticosteroid therapy delayed viral clearance [11, 12], cause a secondary infection [13], and prolonged the duration of hospitalization in COVID-19, middle east respiratory syndrome, and influenza pneumonia [14]. The controversy was principally attributed to the question that it was unclear who can benefit from corticosteroid therapy. Cai et al. found corticosteroid treatment in patients with neutrophil-to-lymphocyte ratio > 6.11 at admission was accompanied by a lower risk of 60-day all-cause mortality. However, they did not systematically screen the factors which might be associated with the efficiency of corticosteroid therapy. And it was unknown whether any other features could predict the efficiency of corticosteroid therapy [15]. Thus, it would make sense to investigate more indicators to guide the corticosteroid use in COVID-19 patients.

Statistical modeling and machine learning algorithms have potential performance in the diagnosis, treatment, prediction of epidemic development, and outcome of COVID-19 patients [16]. There were some models for discovering and repurposing drugs suitable for combating COVID-19 [17, 18]. However, the computational models to help decision-making on precision medication of COVID-19 are currently not available. Given the puzzles of targeting responders to corticosteroid therapy in COVID-19 and the superiority of machine learning, we aim to develop a model to help clinicians identify the patients who will benefit from corticosteroid therapy.

## MATERIALS AND METHODS

### Cohort study design

All COVID-19 patients from two hospitals (Sino-French New City Campus of Tongji Hospital, SF; Optical Valley Campus of Tongji Hospital, OV) between January 27, 2020, and March 30, 2020, were diagnosed according to the Diagnosis and Treatment Protocol of COVID-19 published by the National Health Commission of the People’s Republic of China (Trial Version 7) [19]. The electronic health records (EHR) of diagnosed COVID-19 patients were retrospectively reviewed. Patients who received corticosteroid therapy and were with immune/inflammatory laboratory test results at admission were included in our study. Finally, this multicenter, retrospective study included 666 consecutive COVID-19 patients. Hospitalization time, oxygen supply duration, and outcomes were considered together to evaluate response to corticosteroid therapy since these were well-recognized indicators for judging the efficacy of hormone therapy [15, 20]. Selected patients dying in the hospital were straightforwardly determined as non-response to corticosteroids. Considering the longer hospitalization time and oxygen supply duration in critically ill patients, and to avoid the bias of labeling patients caused by this reason, we divided the remaining patients into general, severe, and critical groups according to the Diagnosis and Treatment Protocol of COVID-19 (Trial Version 7) [19]. In each group, patients with hospitalization time and oxygen supply duration below the median of the population were defined as responders. In contrast, patients with hospitalization time or oxygen supply duration no shorter than the median of the population were defined as non-responders. Besides, patients without receiving oxygen treatment were classified based only on hospitalization time. Thus, all the 666 patients with different severities were classified into responders and non-responders accordingly.

We then randomly partitioned 50% and 50% of participants from SF into training cohort and internal validation cohort, respectively. Participants from OV were used as the external validation cohort. Naturally, there were 268 patients in the training cohort, 267 patients in the internal validation cohort, and 131 patients in the external validation cohort.

### Data preprocessing and feature selection

To enable this model to inform treatment decision-making, we only selected features that are readily available at admission. Since corticosteroids are involved in the regulation of immune and inflammatory factors, a total of 36 candidate immune/inflammatory laboratory tests were collected from EHRs of patients on admission. Trained researchers entered and double-checked the data independently. Features were excluded if ≥20% of values were missing (Supplementary Figure 1A), which resulted in ten features left for model development (Supplementary Figure 1B). Then, we utilized the missForest [21] algorithm to impute the missing values in each hospital respectively (Supplementary Figure 2).

**Figure 1:**
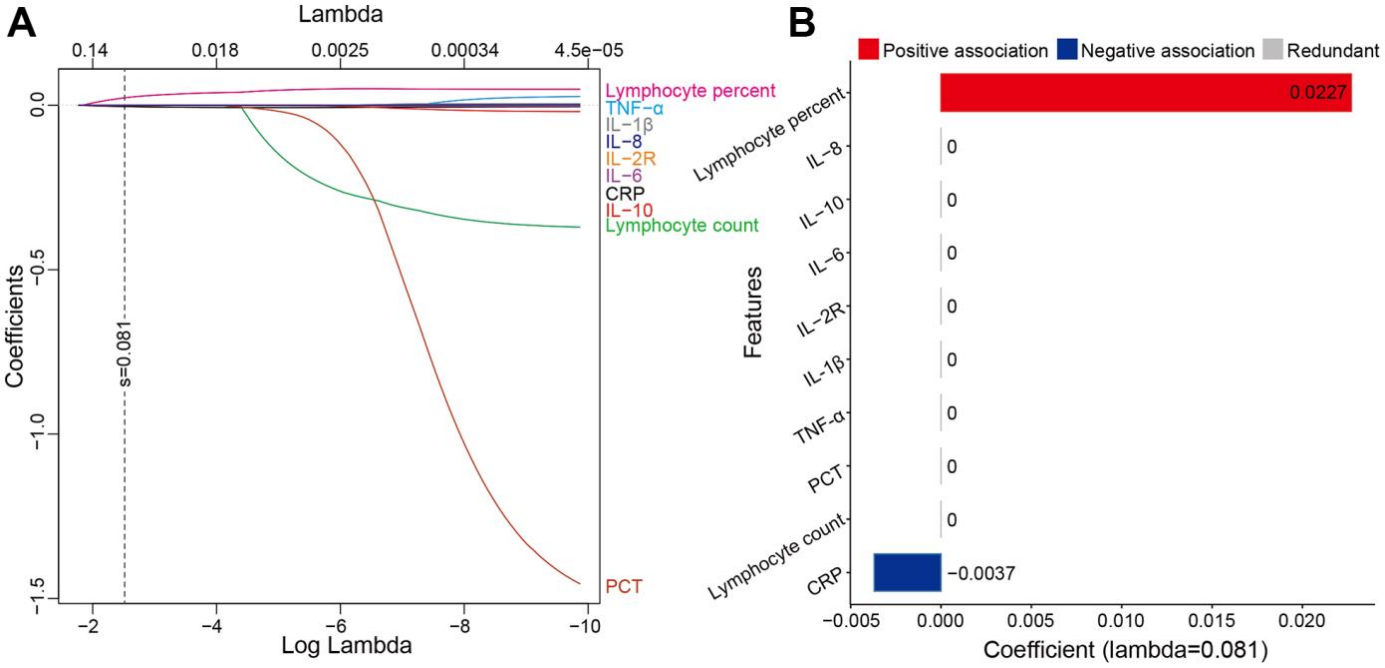
**The features were selected by LASSO.** (**A**) showed LASSO variable trace profiles of the ten features. The vertical dashed line shows the best lambda value (0.081) chosen by tenfold cross-validation. (**B**) showed features with zero coefficient (colored with gray) at lambda = 0.081, was considered less crucial to the patient’s response to corticosteroid therapy and removed by Lasso logistic regression analysis. Features with positive coefficient (colored with red) are regarded as positively associated with response to corticosteroid therapy. Features with negative coefficient (colored with blue) are regarded as negatively associated with response to corticosteroid therapy. Abbreviations: LASSO least absolute shrinkage and selection operator; IL-8 interleukin-8; IL-10 interleukin-10; IL-6 interleukin-6; IL-2R interleukin-2 receptor; IL-1β interleukin-1β; TNF-α tumor necrosis factor α; PCT procalcitonin; CRP C reactive protein.

Features selection aimed to optimize the feature group by identifying the smallest independent set of features with the greatest predictive performance and minimize overfitting. We applied LASSO (Least Absolute Shrinkage and Selection Operator) logistic regression to identify the most informative set of features [22] (Figure 1A). LASSO utilizes the L1 penalty to make the coefficients of weak features turn to zero during fitting [23]. We regarded features with zero coefficients as redundant, and only non-zero coefficient features were included for model training (Figure 1B).

### Model development and validation

As previously demonstrated [24, 25], we fitted the selected features into five computational prediction models, including Logistic Regression (LR), Support Vector Machine (SVM), Gradient Boosted Decision Tree (GBDT), K-Nearest Neighbor (KNN), and Neural Network (NN), to predict whether patients were responsive to corticosteroid therapy. We selected the five models because of their representativity and popularity in EHR prediction. Besides, they are sensitive to different data modalities. For instance, GBDT model is based on the decision tree, where features are merely used to split the node, thus GBDT is not sensitive to scale and distribution of features [26], which is applicable to KNN as well. Therefore, scaling is not required for GBDT and KNN. LR, SVM, and NN train weights by gradient descent, leading to the sensitivity to feature scales. Thus, standardizing data is required to eliminate the differences between features and accelerate the convergence of model [27]. Patients with predictive probability larger or equal to 0.5 were considered as responders to corticosteroid therapy. Otherwise, patients were considered as non-responders. To establish the ensemble model, we tested various combinations of baseline models and discovered that the composition of LR, GBDT, and NN with respective weighted voting 0.1, 0.8, and 0.1 delivered the highest AUC. R library “caret” was adopted for model training and prediction with 10-fold cross-validation. The LR, SVM, GBDT, KNN, and NN were ran with method “glm,” “svmLinearWeights,” “gbm,” “knn,” and “avNNet” with default settings, respectively. Data were standardized before training and testing. We obtained the feature importance of baseline and ensemble model from R package “caret”.

### Statistical analysis

All statistical analysis was conducted with R (version 3.6.2). We obtained the receiver operating characteristics (ROC) curve and AUC analysis with R “pROC” package. We plotted the calibration curve with R “rms” package. We calculated the accuracy (ACC), sensitivity (SE), specificity (SP), positive predictive value (PPV), negative predictive value (NPV), Cohen’s kappa coefficient (Kappa), F1 score, and Brier score with R “caret,” “epiR,” and “rms” packages. We considered P values less than 0.05 as statistically significant. the ninety-five percent confidence interval (CI) was reported if necessary.

### Ethics approval

The study was performed in accordance with the tenets of the Declaration of Helsinki and the Good Clinical Practice principles. This study was approved by the Research Ethics Commission of Tongji Medical College, Huazhong University of Science and Technology (TJIRB20200406) with waived informed consent by the Ethics Commission mentioned above. This study was registered in the Chinese Clinical Trial Registry (ChiCTR2000032161).

### Availability of data and material

Clinical data without names of patients can be requested from the corresponding author by signing Material Transfer Agreement.

## RESULTS

### Baseline characteristics of patients

From 666 patients included in the study, 268, 267, and 131 patients were included in the training, internal validation, and external validation cohort, respectively (Table 1). According to the definitions of responders and non-responders to corticosteroid therapy in this study, the training cohort comprised 103 responders and 165 non-responders; internal validation cohort, 108 responders and 159 non-responders; external validation cohort, 49 responders, and 82 non-responders. The median age in the training cohort, internal validation cohort, and external validation cohort was 64 (54.5–72) years, 64 (52–70.5) years, and 63 (50–70) years, respectively. There were 69 patients, 178 patients, and 96 patients with severe and critical illness in the three cohorts respectively. The median hospitalization time was 23 (16–32) days, 21 (14–28) days, and 19 (11.5–27.5) days, and the median oxygen supply duration was 15 (7–24) days, 14 (6–23), and 11 (4–21) days in the training, internal validation, and external validation cohort, respectively. Finally, 203 (75.75%) patients in the training cohort, 189 (70.79%) patients in the internal validation cohort, and 81 (61.83%) patients in the external validation cohort were discharged from hospitals. Hypertension (40.8%–51.1%) was the most common comorbidity among the selected patients (Table 1). Detailed baseline characteristics of cohorts were presented in Table 1. We also analyzed the baseline characteristics of non-responders and responders to corticosteroid therapy. Except for age, the rate of hypertension and dyspnea, the duration of hospitalization time, and oxygen supply duration, there were no other significantly different characteristics between non-responders and responders. We excluded dead patients when calculating the oxygen supply duration and hospitalization time of non-responders and responders for the fact that we defined dead patients as non-responders without considering the oxygen supply duration and hospitalization time. The details were presented in Supplementary Table 2.

**Table 1:** Baseline clinical characteristics of patients.

**Characteristics**	**Total** **(*N*=666)**	**Training cohort** **(*N*=268)**	**Internal validation cohort (*N*=267)**	**External validation cohort (*N*=131)**
**Age (years),** median (IQR)	64 (54–71)	64 (54–71)	64 (52.5–71)	67 (59–75)
**Sex, *n* (%)**				
Female	295 (44.29)	123 (45.90)	122 (45.69)	50 (38.17)
Male	371 (55.71)	145 (54.10)	145 (54.31)	81 (61.83)
**Hypertension, *n* (%)**	296 (44.44)	120 (44.78)	109 (40.82)	67 (51.15)
**CHD, *n* (%)**	75 (11.26)	22 (8.21)	33 (12.36)	20 (15.27)
**Diabetes, *n* (%)**	128 (19.22)	46 (17.16)	57 (21.35)	25 (19.08)
**COPD, *n* (%)**	11 (1.65)	3 (1.12)	4 (1.50)	4 (3.05)
**CKD, *n* (%)**	13 (1.95)	8 (2.99)	3 (1.12)	2 (1.53)
**Severity, *n* (%)**				
General group	323 (48.50)	199 (75.25)	89 (33.33)	35 (26.72)
Severe and critical group	343 (51.50)	69 (24.75)	178 (66.67)	96 (73.28)
**Fever, *n* (%)**	587 (88.14)	236 (88.06)	246 (92.13)	105 (80.15)
**Cough, *n* (%)**	499 (74.92)	198 (73.88)	195 (73.03)	106 (80.92)
**Dyspnea, *n* (%)**	365 (54.80)	157 (58.58)	141 (52.81)	67 (51.15)
**Sputum, *n* (%)**	268 (40.24)	100 (37.31)	102 (38.20)	66 (50.38)
**Fatigue, *n* (%)**	272 (40.84)	96 (35.82)	130 (48.69)	46 (35.11)
**Diarrhea, *n* (%)**	179 (26.88)	73 (27.24)	81 (30.34)	25 (19.08)
**Myalgia, *n* (%)**	143 (21.47)	54 (20.15)	64 (23.97)	25 (19.08)
**Hospitalization time,** days, median (IQR)	22 (14–30)	23 (16–32)	21 (14–28)	19 (11.5–27.5)
**Oxygen supply duration**, days, median (IQR)	15 (6–23)	15 (7–24)	14 (6–23)	11 (4–21)
**Ventilation model, *n* (%)**				
**Non-invasive ventilation**	514 (82.24%)	214 (85.26%)	205 (81.67%)	95 (77.24%)
**Invasive ventilation**	111 (17.76%)	37 (14.74%)	46 (18.33%)	28 (22.76%)
**Outcomes**				
Discharge	473 (71.02)	203 (75.75)	189 (70.79)	81 (61.83)
Death	193 (28.98)	65 (24.25)	78 (29.21)	50 (38.17)
**Lymphocyte percent (%),** median (IQR)	8.20 (3.30–14.40)	8.75 (4.05–15.03)	7.70 (3.10–13.75)	7.10 (2.93–15.98)
**CRP**				
mg/l, median (IQR)	79.35 (34.13–150.53)	69.30 (31.00–126.20)	87.30 (39.80–160.70)	84.30 (26.58–151.65)
**Response to Corticosteroid therapy, *n* (%)**				
Response	260 (39.03)	103 (38.43)	108 (40.45)	49 (37.40)
Non-response	406 (60.96)	165 (61.57)	159 (59.55)	82 (62.60)

Abbreviation: IQR, interquartile ranges; CHD, coronary heart disease; COPD, chronic obstructive pulmonary disease; CKD, chronic kidney disease; CRP, C reactive protein.

### Data preprocessing and feature selection

We did not include the baselines into features selection for the fact that most of the baselines were not significantly different between responders and non-responders except age (Supplementary Table 2). However, studies have indicated different age groups had different vulnerabilities, immune responses, and inflammatory responses to SARS-CoV-2, which was manifested by the levels of immune/inflammatory features [28–30]. Thus, we did not include age in our prediction model as we have considered the broadest immune/inflammatory features. Finally, 34 included raw candidate immune/inflammatory features, except interferon-γ and interleukin-4 which were not available in selected patients, were shown in Supplementary Table 1. Features with a proportion of missing values greater than or equal to 20% were filtered (Supplementary Figure 1A), resulting in ten features left, including lymphocyte count, lymphocyte percent, tumor necrosis factor α (TNF-α), interleukin-1β (IL-1β), interleukin-2 receptor (IL-2R), interleukin-6 (IL-6), interleukin-8 (IL-8), interleukin-10 (IL-10), C reactive protein (CRP), and procalcitonin (PCT), for further analysis (Supplementary Figure 1B). Features before (Supplementary Figure 2A, 2C) and after (Supplementary Figure 2B, 2D) imputation were provided in SF and OV, respectively.

LASSO logistic regression finally identified two most informative features (lymphocyte percent, CRP) for model development (Figure 1A). Lymphocyte percent was positively correlated (0.0227) with the response to corticosteroid therapy, while CRP was accompanied by a negative correlation (−0.0037) with the response to corticosteroid therapy (Figure 1B). Since lymphocyte percent was an indicator of immune status and CRP was one of the most sensitive markers of inflammation in various diseases [31, 32], it suggested that the predictive markers selected by LASSO were theoretically closely related to corticosteroid therapy.

### Model performance

**Figure 2:**
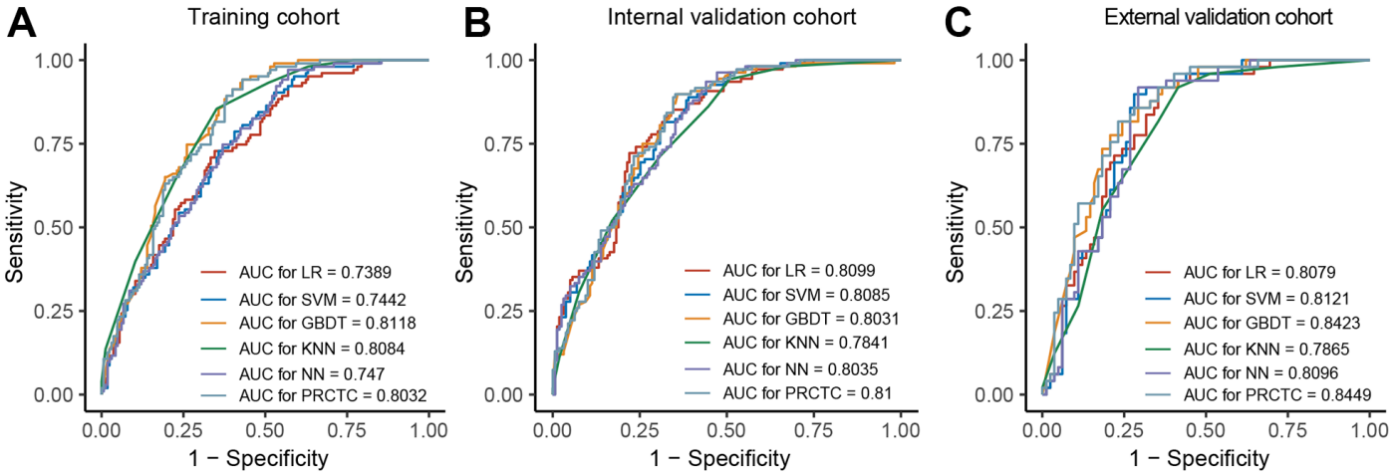
**PRCTC achieved a prompt performance in evaluation on the validation datasets.** (**A**–**C**) showed ROC curve and AUC of SVM, LR, GBDT, KNN, and NN in training cohort, internal validation cohort, and external validation cohort, respectively. Abbreviations: PRCTC, prediction of response to corticosteroid therapy in COVID-19 patients; ROC, receiver operating characteristic curve; AUC, area under the curve; SVM, supported vector machine; LR, logistic regression; GBDT, gradient boosted decision tree; KNN, k-nearest neighbor; NN, neural network.

In general, all five models (LR, SVM, GBDT, KNN, and NN) showed similar and promising corticosteroid therapy response prediction performance across cohorts (Table 2). The AUC was 0.740 with LR, 0.744 with SVM, 0.812 with GBDT, 0.808 with KNN, and 0.747 with NN for the training cohort (Figure 2A). The AUC was 0.810 with LR, 0.809 with SVM, 0.803 with GBDT, 0.784 with KNN, and 0.804 with NN for the internal validation cohort (Figure 2B). The AUC was 0.808 with LR, 0.812 with SVM, 0.842 with GBDT, 0.787 with KNN, and 0.810 with NN for the external validation cohort (Figure 2C). Then we have tried various permutations of the baseline models with different voting weights and found that the ensemble model PRCTC (prediction of response to corticosteroid therapy in COVID-19 patients) derived from GBDT, NN, and LR achieved the best predictive performance. The relative importance of features included in models is shown in Supplementary Figure 3.

**Table 2:** Performance for prediction of response to corticosteroid therapy of models in different cohorts.

	**AUC (95% CI)**	**Accuracy (95% CI)**	**SN** **(95% CI)**	**SP** **(95% CI)**	**PPV** **(95% CI)**	**NPV** **(95% CI)**	**Kappa**	**F1**	**Brier**
**Internal validation cohort**									
LR	0.810 (0.759–0.861)	0.670 (0.611–0.727)	0.444 (0.349–0.543)	0.824 (0.756–0.880)	0.632 (0.513–0.739)	0.686 (0.615–0.751)	0.282	0.522	0.179
SVM	0.809 (0.758–0.859)	0.6854 (0.626–0.741)	0.370 (0.279–0.469)	0.899 (0.842–0.941)	0.714 (0.578–0.827)	0.678 (0.610–0.740)	0.292	0.488	0.188
GBDT	0.803 (0.751–0.855)	0.730 (0.673–0.783)	0.694 (0.598–0.780)	0.755 (0.680–0.819)	0.658 (0.563–0.744)	0.784 (0.711–0.847)	0.445	0.676	0.180
KNN	0.784 (0.731–0.837)	0.704 (0.645–0.758)	0.519 (0.420–0.616)	0.830 (0.763–0.885)	0.675 (0.563–0.774)	0.717 (0.647–0.781)	0.362	0.586	0.192
NN	0.804 (0.753–0.854)	0.700 (0.642–0.755)	0.676 (0.579–0.763)	0.717 (0.640–0.786)	0.619 (0.525–0.707)	0.765 (0.689–0.831)	0.387	0.646	0.180
PRCTC	0.810 (0.760–0.861)	0.738 (0.681–0.790)	0.685 (0.589–0.771)	0.774 (0.701–0.836)	0.673 (0.577–0.759)	0.783 (0.711–0.845)	0.457	0.679	0.177
**External validation cohort**									
LR	0.808 (0.734–0.882)	0.725 (0.640–0.780)	0.571 (0.422–0.712)	0.817 (0.716–0.894)	0.651 (0.491–0.790)	0.761 (0.659–0.846)	0.398	0.609	0.171
SVM	0.812 (0.739–0.885)	0.687 (0.600–0.765)	0.429 (0.288–0.578)	0.842 (0.744–0.913)	0.618 (0.436–0.778)	0.711 (0.611–0.799)	0.288	0.506	0.191
GBDT	0.842 (0.776–0.908)	0.779 (0.698–0.847)	0.776 (0.634–0.882)	0.781 (0.675–0.864)	0.679 (0.540–0.797)	0.853 (0.753– 0.924)	0.541	0.724	0.157
KNN	0.787 (0.710–0.863)	0.718 (0.632–0.793)	0.551 (0.402–0.693)	0.817 (0.716–0.894)	0.643 (0.480–0.785)	0.753 (0.650–0.838)	0.379	0.593	0.183
NN	0.810 (0.736–0.883)	0.733 (0.649–0.806)	0.735 (0.589–0.851)	0.732 (0.622–0.824)	0.621 (0.484–0.745)	0.822 (0.715–0.902)	0.450	0.673	0.163
PRCTC	0.845 (0.779–0.911)	0.771 (0.690–0.840)	0.755 (0.611– 0.867)	0.781 (0.675–0.864)	0.673 (0.533–0.793)	0.842 (0.740–0.916)	0.523	0.712	0.156

Abbreviation: AUC, area under the curve; LR, logistic regression; SVM, supported vector machine; GBDT, gradient boosted decision tree; KNN, k-nearest neighbor; NN, neural network; PRCTC, prediction of response to corticosteroid therapy in COVID-19 patients; SN, sensitivity; SP, specificity; PPV, positive predictive value; NPV, negative predictive value; CI, confidence interval.

For the training cohort, PRCTC displayed an AUC of 0.803 (95%CI 0.752–0.854) in predicting the response to corticosteroid therapy (Figure 2A). For the internal validation cohort, PRCTC achieved an AUC of 0.810 (95%CI 0.760–0.861) to predict the response to corticosteroid therapy in COVID-19 patients with an accuracy of 0.738 (95%CI 0.681–0.790) (Figure 2B). For the external validation cohort, PRCTC demonstrated an AUC of 0.845 (95%CI 0.779–0.911) and an accuracy of 0.771 (95%CI 0.690–0.840) (Figure 2C).

**Figure 3:**
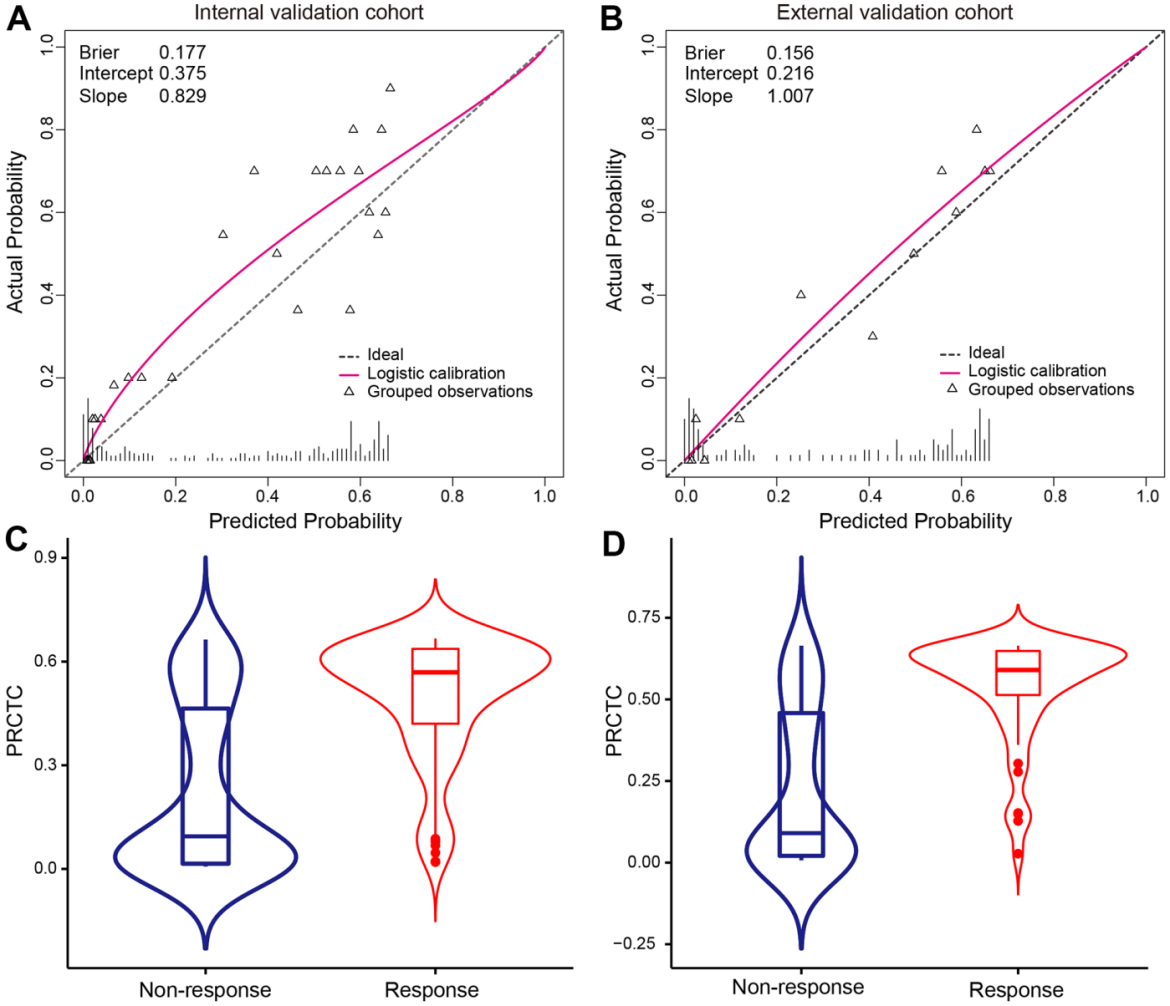
**Calibration curves of PRCTC model were shown in validation cohorts.** Calibration curves of PRCTC model were shown for internal validation cohort (**A**) and external validation cohort (**B**), respectively. The triangle represents the observation group. Each group contained an average of 20 observations. The dashed line is the datum line. The bottom vertical lines refer to the predicted probability distribution. The red curve is the fitted nonparametric calibration curve. PRCTC predicted probability distribution on ground-truth no-response and response patients were shown in internal validation (**C**) and external validation cohort (**D**), respectively. Abbreviations: PRCTC, prediction of response to corticosteroid therapy in COVID-19 patients.

The calibration curves of PRCTC in two validation cohorts were depicted in Figure 3, showing that PRCTC displayed the minimal Brier score of 0.177 (intercept 0.375, slope 0.829) for the internal validation cohort (Figure 3A) and 0.156 (intercept 0.216, slope 1.007) for external validation cohort (Figure 3B). As a result, no further modifications of models were performed. Figure 3C–3D further illustrated the ensemble predicted probability distribution on ground-truth no-response and response patients in internal validation and external validation cohort, respectively.

## DISCUSSION

The management of COVID-19 patients remains the top priority in areas where the virus is raging since vaccinations are likely to take years to reach full coverage and may not protect against new variants. Though corticosteroid has been proved to be one of the few effective drugs for COVID-19 patients, the population who would benefit from corticosteroid therapy remains unclear. The key to solving this clinical challenge is timely and precisely identifying COVID-19 patients who may respond to corticosteroid therapy in the context of limited medical resources. In this study, we developed and validated an ensemble model named PRCTC, derived from LR, GBDT, and NN, which achieved the best performance in prediction among all the integrated models to predict the response to corticosteroid therapy based on lymphocyte percent and CRP at admission. By calculating the inputted values of CRP and lymphocyte percentage, the model will output the probability of response to corticosteroid therapy. Patients with predictive probability larger or equal to 0.5 were considered as responders to corticosteroid therapy. Otherwise, patients were considered as non-responders. Surprisingly, the model performed an AUC of 0.810 in the internal validation cohort and 0.845 in the external validation cohort. With this model, clinicians could make a prompt and precise medical decision on whether to apply corticosteroids to COVID-19 patients and therefore prevent unneeded patients from side effects of corticosteroid therapy.

The two selected features were reliable in this prediction model. Lymphocyte percent and CRP have been reported to be associated with the severity and outcome of COVID-19 [33, 34]. Decreased lymphocyte percent and elevated CRP are universal in patients with COVID-19, especially in critical patients [35]. In this study, increased lymphocyte percent was positively correlated with the response to corticosteroid therapy while elevated CRP was negatively correlated with response to corticosteroid therapy. The results were consistent with the conclusions of previous studies about corticosteroid therapy in critical patients with other diseases. The Adjunctive Corticosteroid Treatment in Critically Ill Patients with Septic Shock (ADRENAL) trial demonstrated that hydrocortisone infusion in sepsis patients subjected to mechanical ventilation did not reduce the mortality compared with that of patients receiving standard care and placebo. What’s more, the mortality in this clinical trial was close to the mortality of critically ill patients with COVID-19 [36, 37]. Some other systematic reviews and meta-analyses also did not recommend corticosteroid use for sepsis [38–40]. Given the fact that the pathophysiology of sepsis, characterized by cytokine release, systemic inflammation, lymphopenia, and following immunosuppression, is similar to that of COVID-19 to a certain extent [41, 42], we have reason to believe the reliability of two selected features in our model. Additionally, Ebisawa et al. uncovered that lower CRP predicted stronger corticosteroid responsiveness in multicentric Castleman’s disease with unknown mechanisms [43]. Considering the immunomodulatory and anti-inflammatory functions of corticosteroid [44], the effects of corticosteroid may depend on the function of immune system. And an extremely high level of CRP usually indicates an extremely dysfunctional immune system that cannot be redressed by corticosteroid. However, the exact mechanism is urgent to be investigated in further study. Interestingly, it is lymphocyte percent but not lymphocyte count that is associated with the response of corticosteroid therapy. Lymphocyte percentage is the proportion of lymphocytes to white blood cells. Its level is determined not only by lymphocyte count, but also by the number of other types of white blood cells such as neutrophil granulocytes, eosinophil granulocytes basophilic granulocytes, and monocyte. Furthermore, due to the individual differences in white blood cell count, lymphocyte percentage is a better indicator of immune status than lymphocyte count. Studies also revealed decreased lymphocyte percentage but not lymphocyte count was an independent poor prognostic factor in advanced cancers [45, 46], which reinforced the superiority of lymphocyte percentage. Based on the results of previous studies above, we believe the two features included in PRCTC are clinically reliable for prediction.

Until December 10, 2021, the highly contagious delta variant of SARS-CoV-2 has quickly spread around the world [47]. Its high contagiousness was mostly attributed to the mutations of spike protein [48]. Though the delta variant has been shown to have a 108% increase in hospitalization risk, 235% increase in ICU admission and 133% higher chance of death than the original variant, the pathophysiology of the delta variant and the original variant was similar, characterized by elevated serum levels of cytokines and decreased the count of lymphocyte, and the status of the disease was reflected on the levels of the immune/inflammatory indicators [49]. Besides, a massive vaccination campaign has begun since December 2020. Several studies have reported various vaccines could decrease the transmission of SARS-CoV-2 among the population and prevent the disease from progressing into critical ill by pre-activating the immune system [50–52]. Though we have no idea how the vaccines impact the immune/inflammatory indicators of patients with COVID-19, its effect on the pre-activation of the immune system is universally recognized. And the effect on the immune system will be manifested by the levels of immune/inflammatory indicators, and these are what we are concerned about during the development of this prediction model. In summary, we think our prediction model still works when confronted with the specific population of COVID-19 patients. Given the fact that the SARS-CoV-2 was sporadic in China now, we have little data for further validation in this study. But our research provided a primary model to do such work in the further large-scale, prospective investigation.

There were several advantages of PRCTC. First, this model could be widely applied with great universality. The two features considered in this model were routinely monitored in the process of management of COVID-19 patients, which meant the selected features were easily accessible, even in community medical institutions. Thus, PRCTC could be widely accepted and applied in hospitals with different levels. More importantly, PRCTC had strong scalability of the application. We adopted various types of classification algorithms in the development of PRCTC, which enabled the model to deal with various and complex data. Based on the inside algorithms of the model, PRCTC could potentially extend to other diseases for patient management, including diagnosis, treatment, and prognostic prediction after fine-tuning.

In addition, to our knowledge, PRCTC is the first model so far to enable decision-making on precise corticosteroid use in COVID-19, though numerous computational prediction models were developed for discovering and repurposing suitable drugs, prediction for disease severity and prognosis in the context of COVID-19 [17, 18, 24, 25]. Zhang et al. built a protein 3D model according to the virus RNA, and then performed a screen of mass chemical compounds to identify protein-ligand interacting pairs [17]; Beck et al. developed an artificial intelligence model to predict binding affinity between antiviral drugs approved by Food and Drug Administration and target proteins [18]. These models predicted potential drugs based on the structure of proteins and drugs but still could not precisely recognize the targeted population. In this study, we firstly established a machine learning model named PRCTC to identify the patients benefitting from corticosteroid therapy by involving lymphocyte percent and CRP, which could help the precise medical decision on corticosteroid use in particular patients.

However, there are still some limitations in our research. First, the model achieved promising but moderate AUCs in the internal and external validation cohorts, which may attribute to the limited number of patients in our study. Second, some immune-inflammatory parameters missing in ≥20% of the population did not enter the training process due to the retrospective nature of the study. Third, the types, dosage, and duration of corticosteroid therapy were not considered in this study, given the limited number of patients in each cohort. Thus, a large-scale, prospective investigation is urgent to be designed to refine our study.

## CONCLUSIONS

To conclude, in this multicenter, retrospective study, PRCTC was proposed with robustness, universality, and scalability that enabled accurately and timely identifying COVID-19 patients benefit from corticosteroid therapy. However, these findings warrant further investigation.

## Supplementary Material

Supplementary Figures

Supplementary Tables
