# Supplementary material for: PRCTC: a machine learning model for prediction of response to corticosteroid therapy in COVID-19 patients
Source: Aging (Albany NY). 2022 Jan 12;14(1):54–72. doi: 10.18632/aging.203819 (PMC8791209; doi:10.18632/aging.203819)
Supplement: Supplementary Figures [file aging-14-203819-s001.pdf]

SUPPLEMENTARY FIGURES

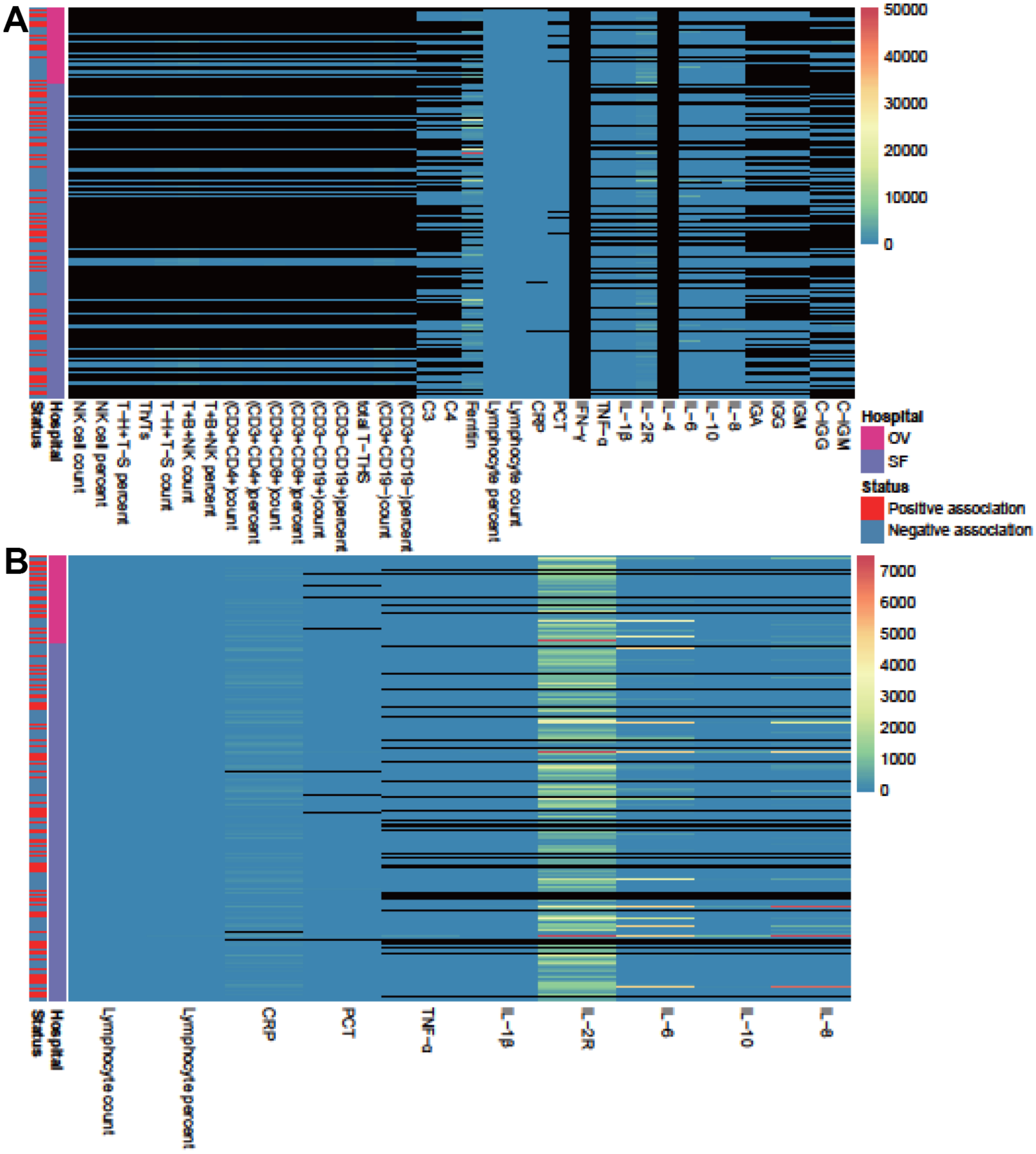

Supplementary Figure 1. Visualization of the denoising and filtering process. (**A**) Heatmap of raw lab test data. (**B**) Heatmap of lab test data after removing lab test features with more than and equal to 20% missing entries across the SF and OV hospitals. Black tiles refer to missing entries. Abbreviations: NK, natural killer; Th, T-helper lymphocyte; Ts, T-suppressor lymphocyte; C3, complement 3; C4, complement 4; CRP, C reactive protein; PCT, procalcitonin; IFN−γ, interferon-γ; TNF-α, tumor necrosis factor α; IL-1β, interleukin-1β; IL-2R, interleukin-2 receptor; IL-4, interleukin-4; IL-6, interleukin-6; IL-8, interleukin-8; IL-10, interleukin-10; IGA, immunoglobulin A; IGG, immunoglobulin G; IGM, immunoglobulin M; C-IGG SARS-CoV-2 specific antibody IgG; C-IGM SARS-CoV-2 specific antibody IgM; SF, Sino-French New City Campus of Tongji Hospital; OV, Optical Valley Campus of Tongji Hospital.

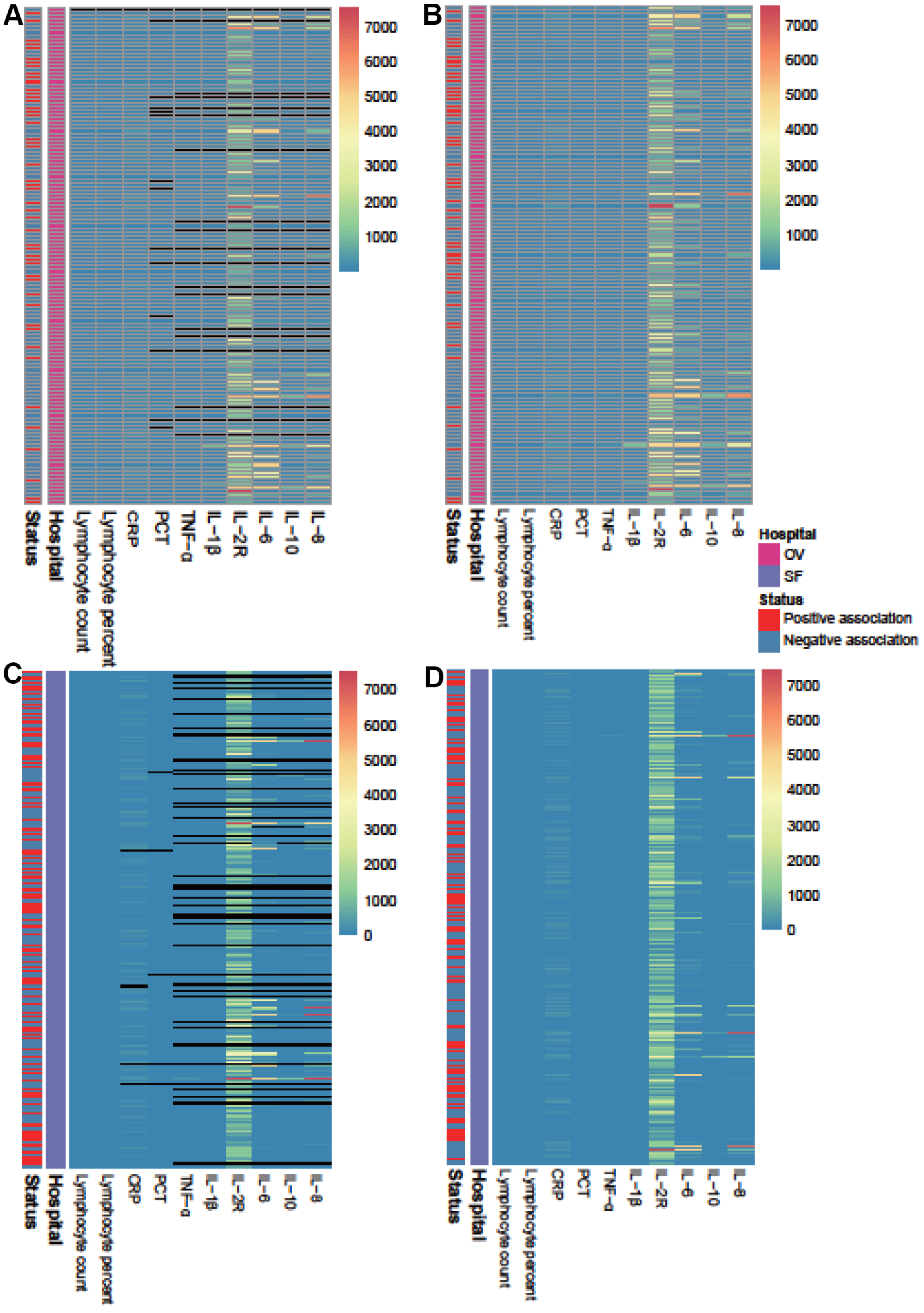

Supplementary Figure 2. Visualization of the imputation process. (**A**, **C**) Heatmap of SF and OV lab test data before imputation. (**B**, **D**) Heatmap of SF and OV lab test data after imputation. Black tiles refer to missing entries. Abbreviations: CRP, C reactive protein; PCT, procalcitonin; TNF-α, tumor necrosis factor α; IL-1β, interleukin-1β; IL-2R, interleukin-2 receptor; IL-6, interleukin-6; IL-8, interleukin-8; IL-10, interleukin-10; SF, Sino-French New City Campus of Tongji Hospital; OV, Optical Valley Campus of Tongji Hospital.

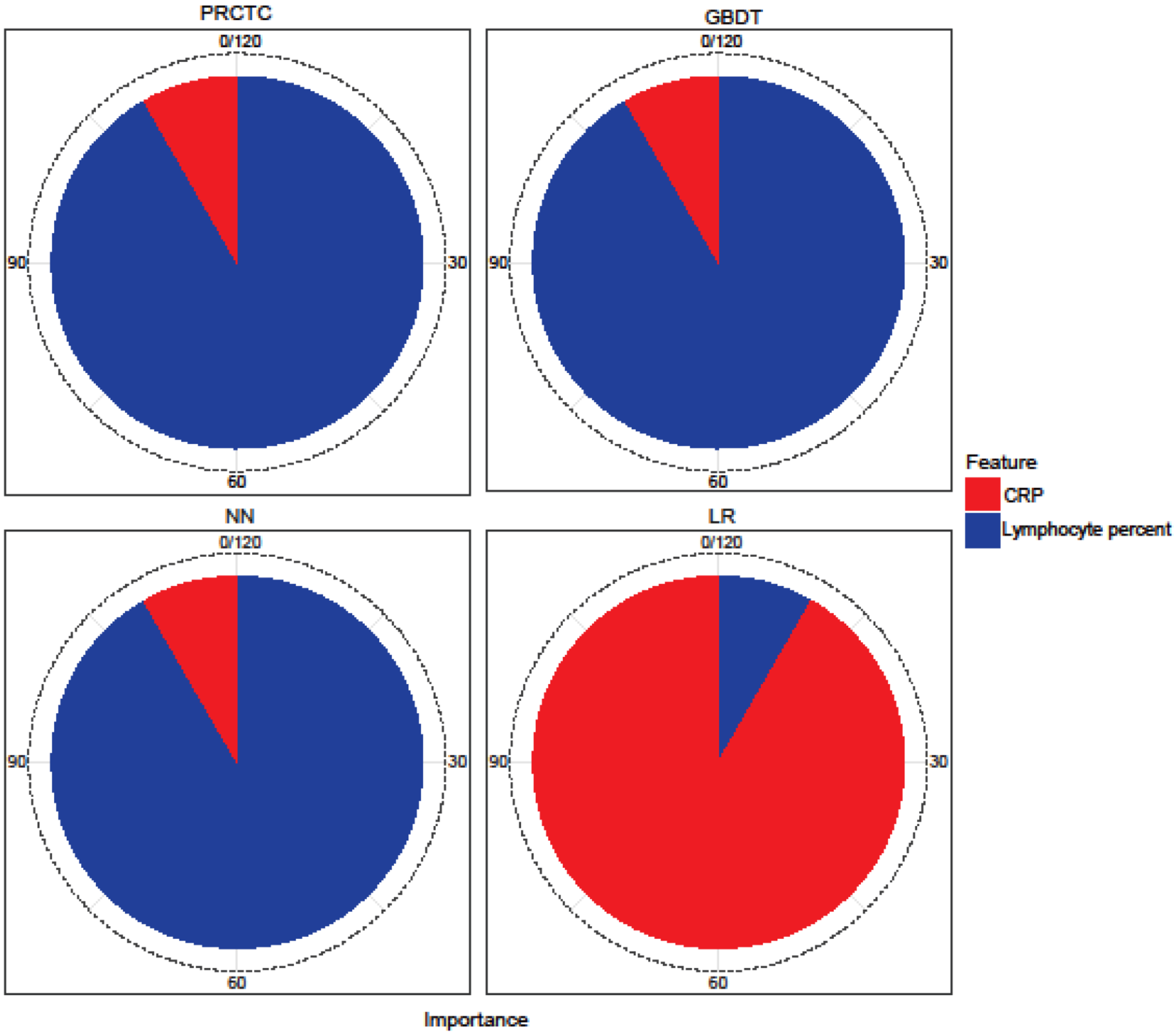

Supplementary Figure 3. The importance of relative features derived from PRCTC, GBDT, NN, and LR model, respectively. Abbreviations: PRCTC, prediction of response to corticosteroid therapy in COVID-19 patients; LR, logistic regression; GBDT, gradient boosted decision tree; NN, neural network.
