# Supplementary material for: PRCTC: a machine learning model for prediction of response to corticosteroid therapy in COVID-19 patients
Source: Aging (Albany NY). 2022 Jan 12;14(1):54–72. doi: 10.18632/aging.203819 (PMC8791209; doi:10.18632/aging.203819)
Supplement: Supplementary Tables [file aging-14-203819-s002.pdf]

SUPPLEMENTARY TABLES

Supplementary Table 1. Data of features before screening in different cohorts.

Features median (IQR)	Total (*N*=666)	Training cohort (*N*=268)	Internal validation cohort (*N*=267)	External validation cohort (*N*=131)
**NK cell count** per µl	111.50 (39.50–193.75)	133.00 (49.00–190.00)	111.50 (45.75–159.50)	101.00 (28.00–199.00)
**NK cell percent, (%)**	9.84 (6.43–17.57)	9.34 (5.77–17.79)	9.84 (7.52–20.73)	10.28 (6.54–15.73)
**(Th+Ts) count** per µl	717.50 (318.25–1045.75)	700.00 (339.75–1185.00)	783.75 (362.38–898.25)	610.00 (284.00–1248.00)
**(Th+Ts) percent, (%)**	65.90 (59.50–74.03)	63.77 (60.71–71.84)	71.25 (61.55–77.71)	66.36 (57.86–71.38)
**Th/Ts**	2.15 (1.31–3.26)	2.50 (1.62–3.26)	1.71 (1.20–2.47)	2.18 (1.44–3.39)
**(T+B+NK) count** per µl	1039.50 (553.75–1520.63)	1151.00 (728.00–1701.00)	1046.00 (620.38–1314.50)	981.50 (472.00–1805.00)
**(T+B+NK) percent, (%)**	99.19 (98.58–99.53)	99.32 (98.69–99.56)	99.03 (98.44–99.37)	99.19 (98.57–99.53)
**(CD3+CD4+) count** per µl	413.50 (191.75–632.00)	415.00 (205.50–736.50)	378.00 (212.75–546.00)	425.00 (185.00–715.00)
**(CD3+CD4+) percent, (%)**	44.81 (37.03–52.36)	45.32 (32.44–50.62)	44.13 (33.13–52.74)	43.09 (39.17–48.86)
**(CD3+CD8+) count** per µl	233.50 (74.50–379.00)	184.00 (78.50–355.00)	260.00 (91.50–353.25)	205.00 (56.00–405.00)
**(CD3+CD8+) percent, (%)**	20.48 (13.57–29.85)	17.93 (14.41–24.36)	24.45 (16.94–31.80)	20.42 (12.23–27.29)
**(CD3-CD19+) count** per µl	126.00 (66.50–218.50)	135.00 (86.00–292.00)	100.50 (46.50–156.50)	145.00 (72.00–266.00)
**(CD3-CD19+) percent, (%)**	15.43 (9.72–26.07)	18.22 (10.47–28.75)	12.07 (7.33–19.02)	17.78 (12.27–22.05)
**Total T-THS**	1.69 (0.68–3.09)	1.60 (0.74–2.69)	1.85 (1.14–3.61)	1.39 (0.47–3.08)
**(CD3+CD19-) count per µl**	682.00 (309.00–1061.50)	680.00 (324.00–1212.00)	704.50 (372.25–925.25)	605.00 (274.00–1263.00)
**(CD3+CD19-) percent, (%)**	69.96 (57.74–78.32)	66.52 (56.80–75.60)	76.08 (62.64–81.80)	70.06 (60.43–75.94)
**C3,** g/L	0.82 (0.69–0.97)	0.83 (0.71–0.98)	0.80 (0.65–0.92)	0.81 (0.64–1.01)
**C4,** g/L	0.21 (0.15–0.28)	0.21 (0.15–0.28)	0.21 (0.14–0.30)	0.19 (0.14–0.26)
**Ferritin** µg/L	957.00 (548.45–1841.70)	917.50 (556.08–1677.95)	968.50 (548.00–1852.50)	1166.20 (503.15–2601.00)
**Lymphocyte count,** per µl	0.60 (0.38–0.93)	0.62 (0.40–0.94)	0.59 (0.35–0.89)	0.60 (0.37–1.02)
**Lymphocyte percent, (%)**	8.20 (3.30–14.40)	8.75 (4.05–15.03)	7.70 (3.10–13.75)	7.10 (2.93–15.98)
**CRP,** mg/L	79.35 (34.13–150.53)	69.30 (31.00–126.20)	87.30 (39.80–160.70)	84.30 (26.58–151.65)
**PCT,** ng/mL	0.11 (0.05–0.45)	0.09 (0.04–0.27)	0.10 (0.05–0.53)	0.19 (0.08–1.48)
**TNF-α,** pg/mL	9.90 (7.50–14.25)	9.50 (7.10–12.60)	9.65 (7.68–13.65)	12.65 (8.43–28.38)
**IL-1β,** pg/mL	5.00	5.00	5.00	5.00

	(5.00–5.45)	(5.00–5.00)	(5.00–5.00)	(5.00–11.08)
**IL-2R,** U/mL	816.00 (494.00–1288.50)	780.00 (458.00–1155.00)	801.00 (473.00–1336.75)	1061.00 (570.75–1653.75)
**IL-6,** pg/mL	24.83 (5.19–89.94)	21.03 (5.01–65.39)	30.38 (5.27–89.78)	27.47 (5.77–276.43)
**IL-8,** pg/mL	20.20 (10.65–54.70)	18.40 (10.00–45.20)	19.15 (10.45–48.75)	30.25 (13.50–110.25)
**IL-10,** pg/mL	6.20 (5.00–13.58)	5.50 (5.00–11.15)	6.90 (5.00–13.95)	6.40 (5.00–20.13)
**IGA,** g/L	2.30 (1.64–2.99)	2.28 (1.63–2.93)	2.23 (1.62–3.09)	2.41 (1.88–3.01)
**IGG,** U/L	12.60 (10.30–5.70)	12.90 (10.33–15.63)	12.40 (10.05–16.08)	12.60 (10.90–14.30)
**IGM,** U/L	0.87 (0.63–1.27)	0.90 (0.63–1.27)	0.82 (0.62–1.29)	0.95 (0.62–1.14)
**C-IGG,** U/L	181.09 (150.49–223.12)	177.83 (146.35–205.31)	181.09 (157.34–222.06)	188.72 (152.19–263.46)
**C-IGM,** U/L	61.71 (26.99–160.88)	55.14 (23.65–148.14)	50.73 (27.57–135.14)	93.02 (44.73–341.40)

Abbreviation: IQR, interquartile ranges; NK, natural killer; Th, T-helper lymphocyte; Ts, T-suppressor lymphocyte; C3, complement 3; C4, complement 4; CRP, C reactive protein; PCT, procalcitonin; TNF-α, tumor necrosis factor α; IL-1β, interleukin-1β; IL-2R, interleukin-2 receptor; IL-6, interleukin-6; IL-8, interleukin-8; IL-10, interleukin-10; IGA, immunoglobulin A; IGG, immunoglobulin G; IGM, immunoglobulin M; C-IGG SARS-CoV-2 specific antibody IgG; C-IGM SARS-CoV-2 specific antibody IgM.

Footnote: the data of interferon-γ and interleukin-4 was missing in all selected patients, thus, we did not make statistics.

Supplementary Table 2. Baseline clinical characteristics of non-responders and responders to corticosteroid therapy.

Characteristics	Non-responders (n=406)	Responders (n=260)	*p*–value
**Age (years),** median (IQR)	66.5 (57–74)	61 (48–67.5)	<0.0001
**Sex, *n* (%)**			0.5397
Female	176 (43.35)	119 (45.77)	
Male	230 (56.65)	141 (54.23)	
**Hypertension, *n* (%)**	198 (48.77)	98 (37.69)	0.005
**CHD, *n* (%)**	53 (13.05)	22 (8.46)	0.0674
**Diabetes, *n* (%)**	81 (19.95)	47 (18.08)	0.5494
**COPD, *n* (%)**	9 (2.22)	2 (0.77)	0.1528
**CKD, *n* (%)**	7 (1.72)	6 (2.31)	0.5954
**Fever, *n* (%)**	359 (88.42)	228 (87.69)	0.7758
**Cough, *n* (%)**	314 (77.34)	185 (71.15)	0.0724
**Dyspnea, *n* (%)**	236 (58.13)	129 (49.62)	0.0313
**Sputum, *n* (%)**	175 (43.10)	93 (35.77)	0.0597
**Fatigue, *n* (%)**	167 (41.13)	105 (40.38)	0.8480
**Diarrhea, *n* (%)**	108 (26.60)	71 (27.31)	0.8409
**Myalgia, *n* (%)**	91 (22.41)	52 (20.00)	0.4593
**Oxygen supply duration[a],** days, median (IQR)	26 (17–32)	14 (6–18)	<0.0001
**Hospitalization time[a],** days, median (IQR)	33 (28–38)	20 (17–24)	<0.0001

Abbreviation: IQR, interquartile ranges; CHD, coronary heart disease; COPD, chronic obstructive pulmonary disease; CKD, chronic kidney disease.

Footnote: [a]We excluded dead patients when calculated the Oxygen supply duration and Hospitalization time of Non-responders and Responders.
